# Hepatitis C virus elimination: time for disruptive innovation

**DOI:** 10.1002/jia2.25360

**Published:** 2019-07-26

**Authors:** Marina B Klein

**Affiliations:** ^1^ Department of Medicine McGill University Health Centre Montreal Canada

**Keywords:** hepatitis C virus, elimination, innovation, direct acting antivirals, screening, prevention, people who inject drugs

As we take stock this World Hepatitis day, a mere 11 years from the World Health Organization's (WHO) 2030 target date for global Hepatitis C Virus (HCV) elimination (a 90% reduction in new diagnoses and 65% reduction in mortality^1^), there is still a very long way to go.

Only nine high‐income countries (Australia, France, Iceland, Italy, Japan, South Korea, Spain, Switzerland and the United Kingdom) are on track to eliminate HCV by 2030 (Figure 1) 2. In fact, 80% of high‐income countries are not on track to meet the WHO targets, and it is estimated that at the current pace of implementation, 67% will not reach elimination targets before 2050, if ever. Although “on‐track” countries include some of those with high burdens of infection such as Italy and Japan, they represent only an estimated 8.5% of the world's 71 million people living with HCV 3. Notably, except Georgia, no country in Eastern Europe or central Asia where HCV infection rates continue to rise unabated among people who inject drugs, is on track.

**Figure 1:**
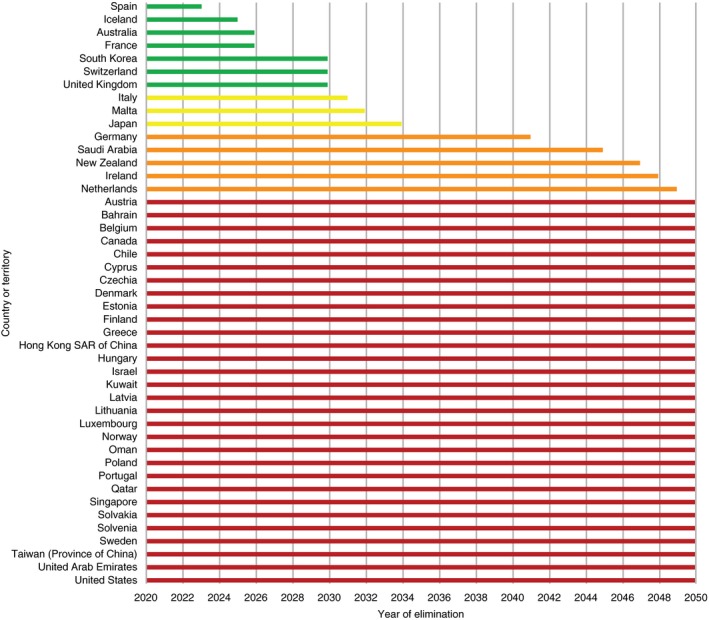
Estimated year of HCV elimination by country.Adapted from: Razavi, H. Global timing of hepatitis C virus elimination: estimating the year countries will achieve the World Health Organization elimination targets. Poster SAT‐260. The International Liver Congress 2019, 15‐19 April, Vienna, Austria..

In low‐ and middle‐income countries (LMICs), there are also some exceptional leaders such as Egypt and Pakistan. However, in far too many countries, the scope of the problem is not even known, let alone efforts at elimination begun 4.

Yes, the elimination goals are ambitious. But let's be clear. We have the tools at our disposal to eliminate HCV as a public health threat – safe, simple curative treatment, accurate diagnostics and knowledge about how transmission can be prevented. The question is, can we mobilize these tools rapidly enough and implement them widely and equitably?

Slow scale‐up will only serve to perpetuate the epidemic over decades and the major health gains associated with HCV treatment will be thwarted. Moreover, the momentum that has built in response to the WHO call to action will be squandered as other important health, social and environmental needs inevitably eclipse this cause.

Only rapid action will yield measurable impact in the short term – modelling predicts greater benefit in prevention of new infections and reductions in decompensated cirrhosis and liver‐related deaths will be realized with rapid scale‐up of prevention, treatment and screening measures 5, 6, 7. Indeed, real world evidence is beginning to confirm this modelling with tangible gains in life expectancy, reduced hospitalizations, liver transplantation and lower rates of new infections 8, 9. As a result, significant savings in health costs are projected. This is a message that policy makers need to hear.

So, what can we learn from the countries that are well along the road to success? These countries uniformly have in common two things: engagement at the highest political level and well designed and executed national action plans.

It is not necessary, however, to wait for a top down approach. Even in the absence of high‐level political commitment, country specific strategies can be developed. An excellent example of this is Canada's recently published “Blueprint to Inform Hepatitis C Elimination Efforts in Canada” 7. It was developed through a consultative and inclusive process involving researchers, clinicians, policy makers, community‐based organizations and people with lived experience. The Blueprint lays out specific targets, some more ambitious than those of the WHO. Most importantly, it is focused on the needs of priority populations – those who experience a disproportionate burden of HCV or who have challenges accessing HCV care and services. It offers options that can be tailored to different contexts and cultures, and is truly a model of how to advance the elimination agenda when government leadership is lacking and jurisdictional and social barriers are entrenched.

But action plans are only the beginning.

Digging deeper, a key feature of successful elimination programmes has been disruption in the normal way of doing business. Disruptive innovation, a business concept coined by Clayton Christensen in 1995, refers to a product that takes root initially at the bottom of a market and then relentlessly moves up, eventually displacing established competitors 10. Generic direct‐acting antivirals (DAAs), by forcing down all DAA prices, have been truly disruptive serving to catalyse elimination programmes worldwide. Indeed, the impact of generic DAAs has had much broader effects by rapidly driving down the cost of diagnostic tests. These changing market forces may ultimately have a lasting impact on all patented medications and diagnostics making health care more equitable.

Rapidly falling drug prices are the result of many forces and actors and are the essential third pillar to successful elimination in on‐track countries. Voluntary and compulsory licenses, patent opposition, joint procurement and personal importation schemes have been some of the effective strategies. A 12‐week generic pan‐genotypic treatment is now as low as $84 in Egypt, $60 in Rwanda and $39 in India – much lower than the floor price of $200 initially predicted 11. Diagnostic tests costs have likewise have fallen from >$100 to $1 for a rapid antibody test and $10 for HCV RNA testing. Compare this to treatment costs of over $40,000 in the US 12. Prices also can come down in high‐income settings. Australia successfully negotiated a volume‐based, risk‐sharing deal with pharmaceutical companies that have allowed them to treat over 80,000 people with HCV for an estimated $8000 per treatment course 13. Italy and Spain have similarly procured DAAs at €8000 per course 13.

But perhaps most disruptive has been the progress made in LMICs in HCV treatment programmes more broadly. Indeed, the successful backward transfer of elimination approaches from LMICs to high‐income settings would be a truly revolutionary impact of DAAs. A good illustration is the Clinton Health Access Initiative Quick Start model 4. This programme, launched in 2016 in Ethiopia, Indonesia, Myanmar, Nigeria, Rwanda and Vietnam with very modest investment, focuses on programme simplification and market shaping to reduce commodity pricing. It includes fast track registration of quality‐assured products, pricing negotiations for both diagnostics and drugs, simplification of national diagnostic and treatment algorithms, decentralized public health approaches to care delivery and leveraging existing HIV care infrastructure.

So political action can be leveraged, costs can be contained and service delivery can change, rapidly, and in a variety of very challenging settings. However, elimination efforts have been very slow in most high‐income settings despite their greater resources. Fragmented health systems and multiple payers result in the inability to innovate rapidly. Lack of transparency in drug pricing and a strong pharmaceutical lobby has kept drug costs high. Siloed health budgets and the reluctance to relinquish antiquated models of specialist based care delivery additionally contribute to a state of inertia in health systems 14.

Governments have bulk buying power, and in the face of major public health crises, can leverage it to negotiate lower prices if they so choose 15. High‐income countries need to capitalize on this to a lower drug and diagnostic costs for diseases with major public health impact – not just for HCV. It is a matter of the very sustainability of healthcare.

Despite progress on many fronts, there are remaining challenges in need of disruptive solutions, even in on track countries. Screening, case finding and linkage to care in populations that have been disenfranchised from health systems remains difficult and has emerged as the major road block to finalizing elimination. As more and more people are cured, cases will be harder and harder to find. Re‐infections will need to be detected and treated early to avoid new outbreaks. This will be particularly important so as not to backslide on the major progress towards elimination that has been made among certain key populations, such as men who have sex with men in Europe 16, 17. Diagnostic testing algorithms will therefore need to change. We will have to move to primary screening with antigen or RNA‐based testing rather than antibody testing. Only then will rapid, point‐of‐care technologies aimed at diagnosing active infections become attractive to developers and costs come down.

Stigma persists and is a barrier for people to seek prevention and treatment services. In an ideal world, curative therapy should normalize HCV infection. Unfortunately, restrictive and punitive drug policies serve to marginalize those bearing the greatest burden of infection. There are many parallel harm reduction innovations emerging such as supervised injection sites 18, prescription heroin 19, and even needle and opioid dispensing machines 20 that if scaled up and paired with elimination efforts could reduce overdoses and help disrupt HCV transmission. Decentralized, destigmatized community‐based models of service delivery tailored to the needs of specific populations are also urgently required.

Several countries have shown us that HCV elimination is possible in both high‐income and low‐income settings. We need to follow and build on their lead. If we succeed in this endeavour, we will not only reduce suffering, save millions of lives and billions in health costs, the resulting disruptive innovations will fundamentally change the way healthcare is delivered to all.

## Competing interests

MBK received research grants for investigator‐initiated trials from Merck, Gilead and ViiV Healthcare; consulting fees from Merck, Gilead and AbbVie. MBK is also supported by a Tier 1 Canada Research Chair.

## Authors’ contributions

MBK was the sole contributor to the preparation of the manuscript.
